# Enhanced Self-Efficacy and Behavioral Changes Among Patients With Diabetes: Cloud-Based Mobile Health Platform and Mobile App Service

**DOI:** 10.2196/11017

**Published:** 2019-05-10

**Authors:** Dyna YP Chao, Tom MY Lin, Wen-Ya Ma

**Affiliations:** 1 Healthcare Solution Center Health Inventor of Taipei Taipei City Taiwan; 2 Graduate Institute of Management National Taiwan University of Science and Technology Taipei City Taiwan; 3 Department of Metabolism Cardinal Tien Hospital New Taipei City Taiwan

**Keywords:** type 2 diabetes mellitus, self-management, health literacy, patient engagement, intervention, word-of-mouth

## Abstract

**Background:**

The prevalence of chronic disease is increasing rapidly. Health promotion models have shifted toward patient-centered care and self-efficacy. Devices and mobile app in the Internet of Things (IoT) have become critical self-management tools for collecting and analyzing personal data to improve individual health outcomes. However, the precise effects of Web-based interventions on self-efficacy and the related motivation factors behind individuals’ behavioral changes have not been determined.

**Objective:**

The objective of this study was to gain insight into patients' self-efficacy with newly diagnosed diabetes (type 2 diabetes mellitus) and analyze the association of patient-centered health promotion behavior and to examine the implications of the results for IoT and mobile health mobile app features.

**Methods:**

The study used data from the electronic health database (n=3128). An experimental design (n=121) and randomized controlled trials were employed to determine patient preferences in the health promotion program (n=62) and mobile self-management education (n=28). The transtheoretical model was used as a framework for observing self-management behavior for the improvement of individual health, and the theory of planned behavior was used to evaluate personal goals, execution, outcome, and personal preferences. A mobile app was used to determine individualized health promotion interventions and to apply these interventions to improve patients’ self-management and self-efficacy.

**Results:**

Mobile questionnaires were administered for pre- and postintervention assessment through mobile app. A dynamic questionnaire allocation method was used to follow up and monitor patient behavioral changes in the subsequent 6 to 18 months. Participants at a high risk of problems related to blood pressure (systolic blood pressure ≥120 mm Hg) and body mass index (≥23 kg/m^2^) indicated high motivation to change and to achieve high scores in the self-care knowledge assessment (n=49, 95% CI −0.26% to −0.24%, *P*=.052). The associated clinical outcomes in the case group with the mobile-based intervention were slightly better than in the control group (glycated hemoglobin mean −1.25%, 95% CI 6.36 to 7.47, *P*=.002). In addition, 86% (42/49) of the participants improved their health knowledge through the mobile-based app and information and communications technology. The behavior-change compliance rate was higher among the women than among the men. In addition, the personal characteristics of steadiness and dominance corresponded with a higher compliance rate in the dietary and wellness intervention (83%, 81/98). Most participants (71%, 70/98) also increased their attention to healthy eating, being active, and monitoring their condition (30% 21/70, 21% 15/70, and 20% 14/70, respectively).

**Conclusions:**

The overall compliance rate was discovered to be higher after the mobile app–based health intervention. Various intervention strategies based on patient characteristics, health care–related word-of-mouth communication, and social media may be used to increase self-efficacy and improve clinical outcomes. Additional research should be conducted to determine the most influential factors and the most effective adherence management techniques.

## Introduction

### Financial Burden

In China, noncommunicable chronic diseases not only are a critical health problem but also impose a major economic burden. Globally, diabetes mellitus (DM) has been diagnosed in 415 million people, and DM and its complications are a global health emergency, accounting for 12% of global health expenditure [1]. The MOST survey revealed that 11.6% of adults in China (approximately 114 million people) have received DM diagnoses. The prevalence of DM has resulted in a substantial financial burden on medical systems, families, and societies. DM is more prevalent in older age groups and among people living in economically developed regions. Overall, DM-related expenditure in 2013 was approximately US $548 billion, accounting for 10.8% of total health expenditure worldwide. DM-related expenditure is expected to exceed US $627 billion by 2035 [2,3].

### Information Technology Transformation

Self-management has become a critical approach for improving health outcomes among patients with DM, and market demand has emerged for related innovations in information technology (IT). Specifically, patients have expressed interest in cloud-based Internet of Things (IoT) measurement devices to record various types of structured and unstructured data (eg, blood glucose, blood pressure, body mass index, body fat, sleep quality, fatigue [4], depressive symptoms, working pressure, and social engagement frequency) [5-7]. Providers hope to integrate patient data into records of patient history, which can be used to derive engagement models of health promotion for increasing patient motivation and adherence [7-9].

The use of mobile health apps as a lifestyle intervention for improving diabetes outcomes has become increasingly common. In 1 study, patients who underwent this intervention exhibited continual improvement in glycemic control compared with baseline values for the duration of the program (with approximately 50% of participants losing at least 5% of their body weight over 6 months). However, the benefits of the transtheoretical model (TTM) [8,10] and behavioral change [4] were not sustained above the control group setting at 3 months after completion of the program [11].

### Patient-Centered Approaches

Patients with diabetes should be encouraged to change their lifestyles and synchronize their personal data recorded inside and outside of hospitals [12]. Thus, patient-centered approaches should be developed for new dynamic behavioral engagement models [13] and as enhancements to existing health promotion models. Personalized approaches should focus on transtheoretical methods as well as patients’ self-management skills, wellness knowledge [9,14], education, psychosocial assessments, satisfaction, and rate of adherence with treatment [6,15].

Adherence exhibits significant beneficial effects on fatigue and social interaction for insulin-using patients with diabetes, emotional well-being for patients with myocardial infarction [4], and glycated hemoglobin (HbA_1c_) for patients with myocardial infarction and diabetes. In addition, researchers have indicated that higher rates of adherence (especially to diet-related recommendations) are associated with improved health outcomes among insulin-using patients with diabetes [11,16].

Observational studies have reported that educational programs for patients with diabetes do not succeed unless they are intensive and continued over a long period with monitoring of behavioral changes and interventions [17-20].

The aim of this study was to identify the personalized influence factors of goal setting, self-execution activities, and self-efficacy for patients with type 2 diabetes. In addition, we aimed to analyze the association of the identified factors with patient-centered health promotion behavior and to encourage patients to engage in electronic word-of-mouth communication [21,22] to motivate their therapy partners [23,24].

## Methods

### Overview

In this study, we developed a cloud-based interactive health care management mobile app, interactive personalized management framework (IPMF), and adopted a platform as a service tier. The platform integrates and displays patient information on a 360-degree dashboard. The dashboard may be used to monitor patient health status and facilitate engagement with physicians. Interactive wellness education is also offered through the mobile-based app and cloud-based interactive service platform [16,25-27]. In addition to genetic and environmental factors, patient behavior is a critical factor associated with chronic disease. The effectiveness of disease management depends not only on patients’ clinical status but also on their lifestyle, disease knowledge, health literacy, beliefs, cognitive state, behavior changes, and emotional state [28-32].

Previous studies have demonstrated that health educators and care managers must understand a patient’s disease literacy level, personal characteristics, and readiness for action to determine effective treatment [1,33]. Care providers should create a service environment that enables patients to adopt appropriate behavioral changes [34,35].

**Figure 1:**
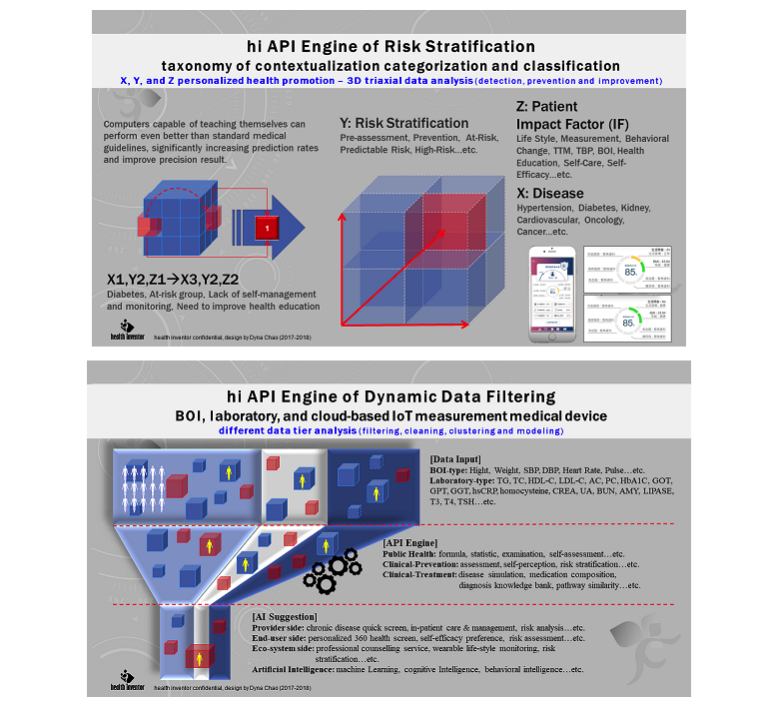
360-Degree health management dashboard displaying three-dimensional triaxial data analysis of IPMF. x: disease, y: level, and z: patient impact factor. API: application programming interface; BOI: body of information; hi: health inventor; IoT: Internet of Things.

Hence, in the developed system, the application programming interface engine uses a three-dimensional analysis tool to aggregate individual patient data and health status. The x-axis denotes disease type and indicates undiagnosed but high-risk and at-risk status, early diagnosed disease, self-perceived symptoms, and diagnosed disease. The y-axis denotes the severity of each symptom. Finally, the z-axis mainly displays the factors that may influence patients’ health in daily life, including family history, education, economic situation, lifestyle, and stress (see Figure 1).

### Methodology

This study used the TTM under the theory of planned behavior to monitor patients’ behavioral changes during the preintervention assessment, follow-up, and postintervention assessment periods [1,5].

Studies have indicated that IT and IoT measurement devices are valuable means of supporting disease management [9,33]. Studies have also reported the use of these technologies in the provision of evidence-based health education tailored to individual patients [16,36]. However, most relevant transtheoretical studies have not analyzed patients' self-knowledge or the effectiveness of patients’ self-care behavior for health management. Specifically, studies have not compared the results of health knowledgeability analyses with continuous data on personal health measurement data and health outcome.

### Recruitment

#### Inclusion and Exclusion

The purpose of this research was to assess the influence of patient insight on adherence to self-management recommendations for patients with new diagnoses and who were at high risk of additional health problems. Accordingly, patients with the following characteristics were eligible to enroll: (1) type 2 DM (T2D) diagnosis received within the prior 3 months, (2) HbA_1c_ % greater than 5.4%, (3) oral glucose tolerance test result greater than 140 mg/dL, and (4) capable to use mobile-based and IT interventions. Patients with DM were invited to participate in the study group based on their disease knowledge and self-management. For the assessment of self-efficacy and cognitive behavioral change, a suitable sample size was determined using the estimated SD of change in self-knowledgeable score versus body weight and HbA_1c_. With an SD of alpha of .05, we estimated that a final sample of 120 participants would provide 85% power to reveal a minimum detectable difference in changes.

#### Randomization and Eligibility

**Figure 2:**
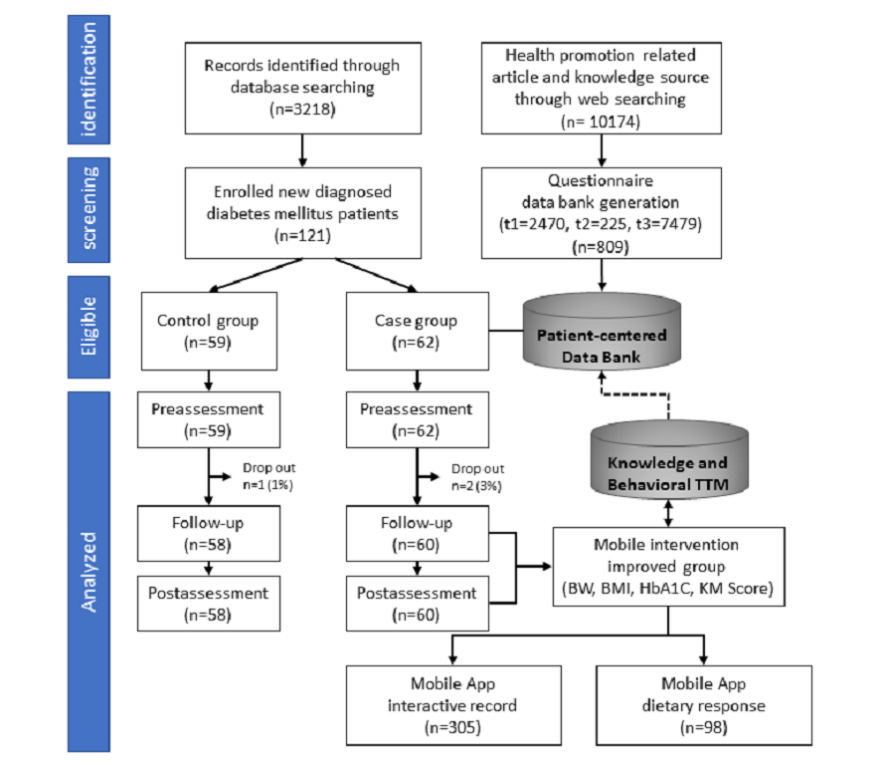
Study enrollment criteria and selection flow. BMI: body mass index; BW: body weight; HbA_1c_: glycated hemoglobin; KM: knowledge management; TTM: transtheoretical model.

In total, 3218 patients with diabetes were identified through a database. These patients were assessed according to the inclusion and exclusion criteria, and ultimately 121 patients (n=121) signed forms of consent and joined the study within 3 to 6 months of communication. Participants were stratified according to age and sex and were then randomly assigned to the case or control group. Case-group patients participated (n=62) in a 1-hour training and employed the IPMF system when they visited outpatient departments. Control-group patients (n=59) were provided with traditional care without support from IT, IoT measurement devices, or the mobile-based app system (see Figure 2).

#### Asian Race or Ethnic Lifestyle

All patients provided informed consent and were interviewed by a professional team comprising a physician, health educator, nutritionist, care manager, and service consultant. All recruited patients were provided with regular clinical advice and medication.

To test the effectiveness of the IPMF and mobile-based interactive results, especially in preferred food style, eating methods, nutrition, and cooking habits, the intervention behavior data bank collected in Taiwan and China. By considering lifestyle and eating habits, this study focused on Taiwan (n=3218) and lifestyle knowledge bank conducted in both China and Taiwan (n=10,174).

### Process and Application Features

**Figure 3:**
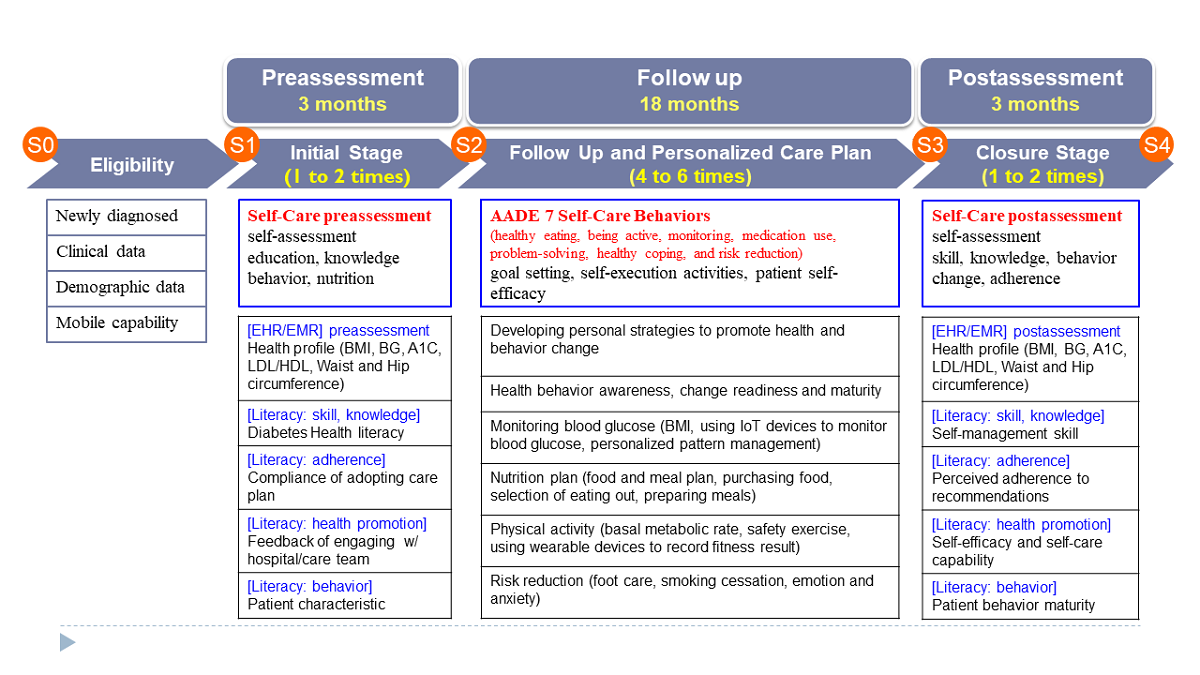
Research design (initial stage, follow-up, and closure). A1C: HbA_1c_ (glycated hemoglobin); AADE: American Association of Diabetes Educators; BG: blood glucose; BMI: body mass index; HDL: high-density lipoprotein; IoT: Internet of Things; LDL: low-density lipoprotein.

The investigation comprised beginning (3 months), follow-up (18 months), and closure phases (3 months). Each outpatient had a different return visit time; therefore, for preassessment, the participants completed self-assessments using the mobile-based app throughout the initial phase (the open period was from months 1-6). Subsequently, each patient collaborated with a physician to set a personal goal and define a health program for the follow-up phase (from months 6-18). By the time they had completed 4 to 6 visits to the outpatient department, each participant had finished the postassessment of the closure phase (from months 18-24). To integrate behavioral activities into the health promotion service flow, the mobile apps were provided to the participants through a mobile tablet while they were waiting to see a doctor or health educator (see Figure 3).

**Figure 4:**
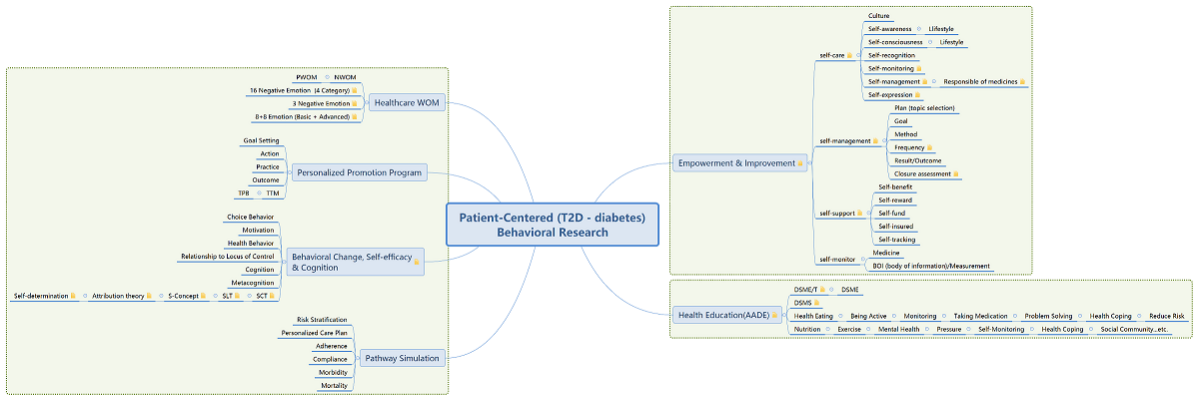
Patient-centered application framework and features. T2D: type 2 DM; TTM: transtheoretical model; WOM: word-of-mouth NWOM: negative word-of-mouth; PWOM: positive word-of-mouth; S-Concept: self-concept theory; SCT: social cognitive theory; SLT: social learning theory; TPB: teory of planned behavior.

Mobile tablets, such as iPads and mobile phone, also served as supporting tools for physicians and health educators to access patients’ integrated information. Patient compliance data were collected through the interface of the IPMF as the patient entered the start phase (1-6 months) for preintervention assessment and the closure phase (18-24 months) later for postintervention assessment. The patient self-assessment score was a taxonomy reference. The behaviors outlined in the American Association of Diabetes Educators (AADE) 7 Self-Care Behaviors program [14,34,37] were used as indicators. These behaviors comprise healthy eating, being active, blood-glucose and blood-pressure monitoring, medication use, problem-solving, use of healthy coping strategies, and risk reduction (see Figure 4).

Patient demographic data that were relevant to health promotion factors were provided by the hospitals providing care and inputted into the IPMF. Participants then received interventional DM education about the American Association of Diabetes Educators 7 Self-Care Behaviors (AADE7). Physicians and health educators conducted educational interventions according to patient’s interests and specific requirements to enhance the patient’s self-management abilities. The interactive mobile physician dashboard system improved patients’ willingness to engage in continual health education.

#### Application Framework

The proposed framework was used to construct a DM education service platform that integrated the collected information. The platform also served as an automatic knowledge bank for aggregating patient learning behavior. The developed framework may be used to help physicians and health educators understand patients’ specific needs and areas for improvement. The IPMF model not only aggregates information from clinical databases but also involves a questionnaire generation mechanism and knowledge bank feature for collecting information regarding patients’ DM literature, DM-related beliefs and emotions, diet, glucose self-monitoring, self-motivation, self-education, and the influence of disease on patient productivity.

The developed mobile app was to analyze patient lifestyles and compliance tendencies and to use a knowledge simulation platform to determine factors for improvement. Analysis of these factors was used to improve clinical outcomes and to enhance patient-physician engagement [38-40].

#### Self-Management Preference

According to the frequency of their access of mobile app features, patients exhibit individual preferences for self-care management, and these preferences influence overall compliance and clinical outcomes. The system collected data regarding the heterogeneity of patients’ lifestyles, knowledge and feelings about diseases, states of action readiness, self-motivation elements of health improvement, and risk conditions.

#### Intervention

An IPMF interactive system was used to observe the patient-insight factor and encompassed disease history, family history, and relevant characteristics. The system was presented through a 360-degree dashboard and provided personalized patient intervention suggestions to physicians and health educators.

#### Influence Factor

Patient characteristics and self-knowledge may have influenced compliance. Moreover, age, sex, education level, and income level affected patient acceptance of IT applications. The system identified the influential factors and their associations with compliance.

### Data Collection Method

#### Interactive Patient Assessment System (Mobile App, iPad)

**Figure 5:**
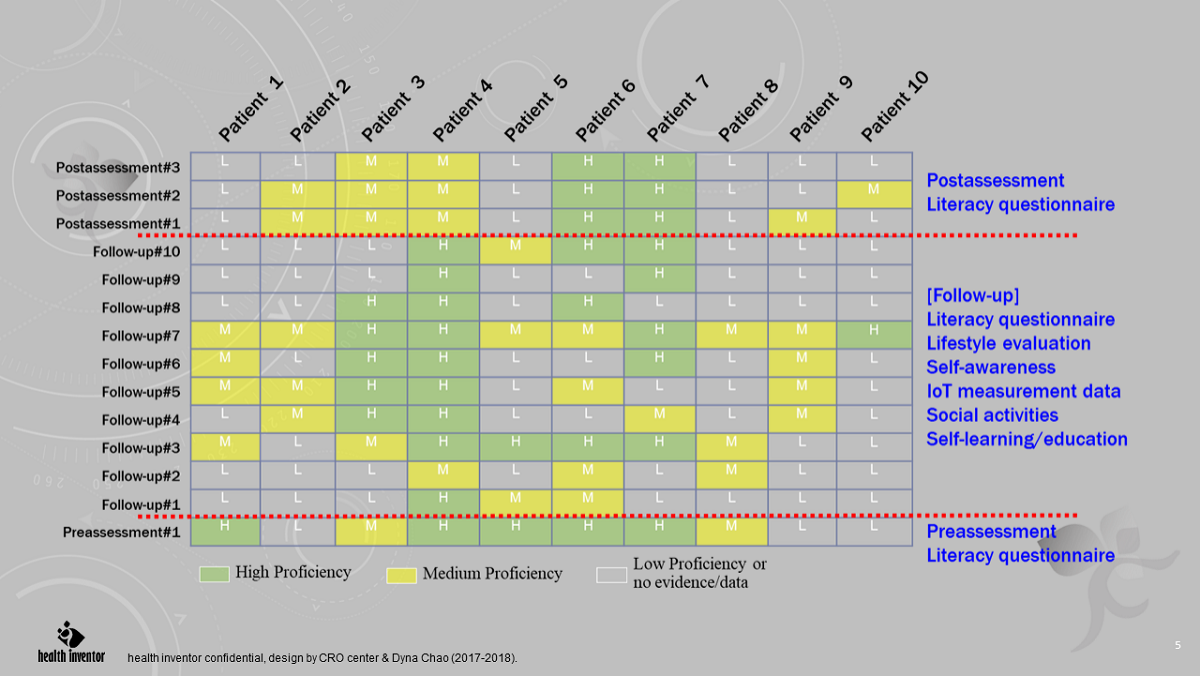
Behavior maturity management (transtheoretical model): pre- and postassessment. IoT: Internet of Things.

**Figure 6:**
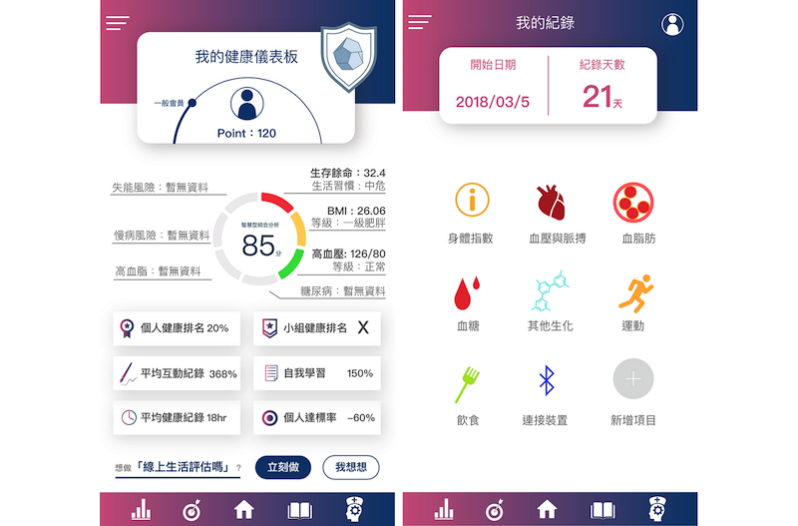
Interactive patient pre- and postassessment.

In the study, patients’ health status, lifestyle, behavior change, and treatment preferences were collected through TTM framework for observing self-management behavior (see Figure 5). Pre- and postintervention assessments via mobile app were conducted (see Figure 6). Patient feedback was stored in a cloud-based IPMF platform and was used to determine factors that contributed to sustainable behavior changes.

#### Patient Readiness Evaluation System (Mobile App, iPad)

**Figure 7:**
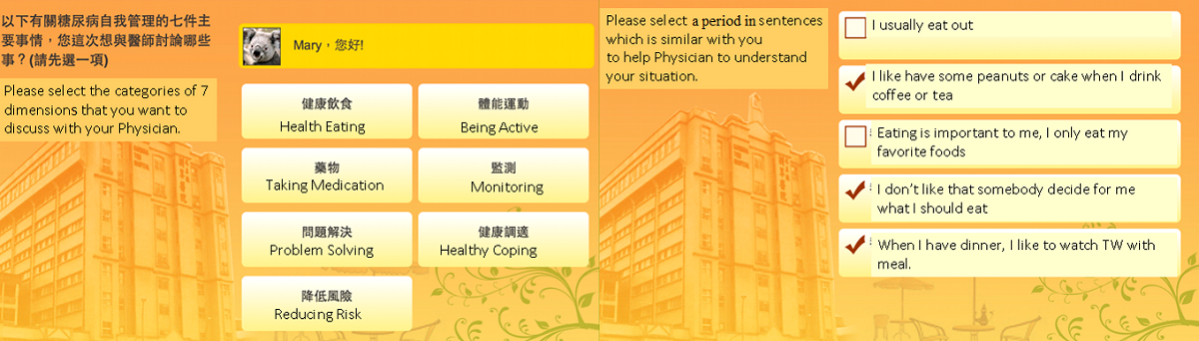
The American Association of Diabetes Educators diabetes self-care behavior quiz.

As described, the AADE7 Self-Care Behaviors are critical to the management of DM (see Figure 7). Thus, in the study, the system collected the information of patients with T2D regarding healthy eating, physical activity, blood glucose and blood pressure monitoring, medication use, problem solving, risk reduction, and use of healthy coping strategies. The collected information was used to evaluate patients’ mental readiness regarding the management of DM. After the readiness evaluation, patient score and disease level were transmitted to a physician dashboard for the next intervention stage.

#### Interactive Physician Dashboard System (Mobile App, iPad)

**Figure 8:**
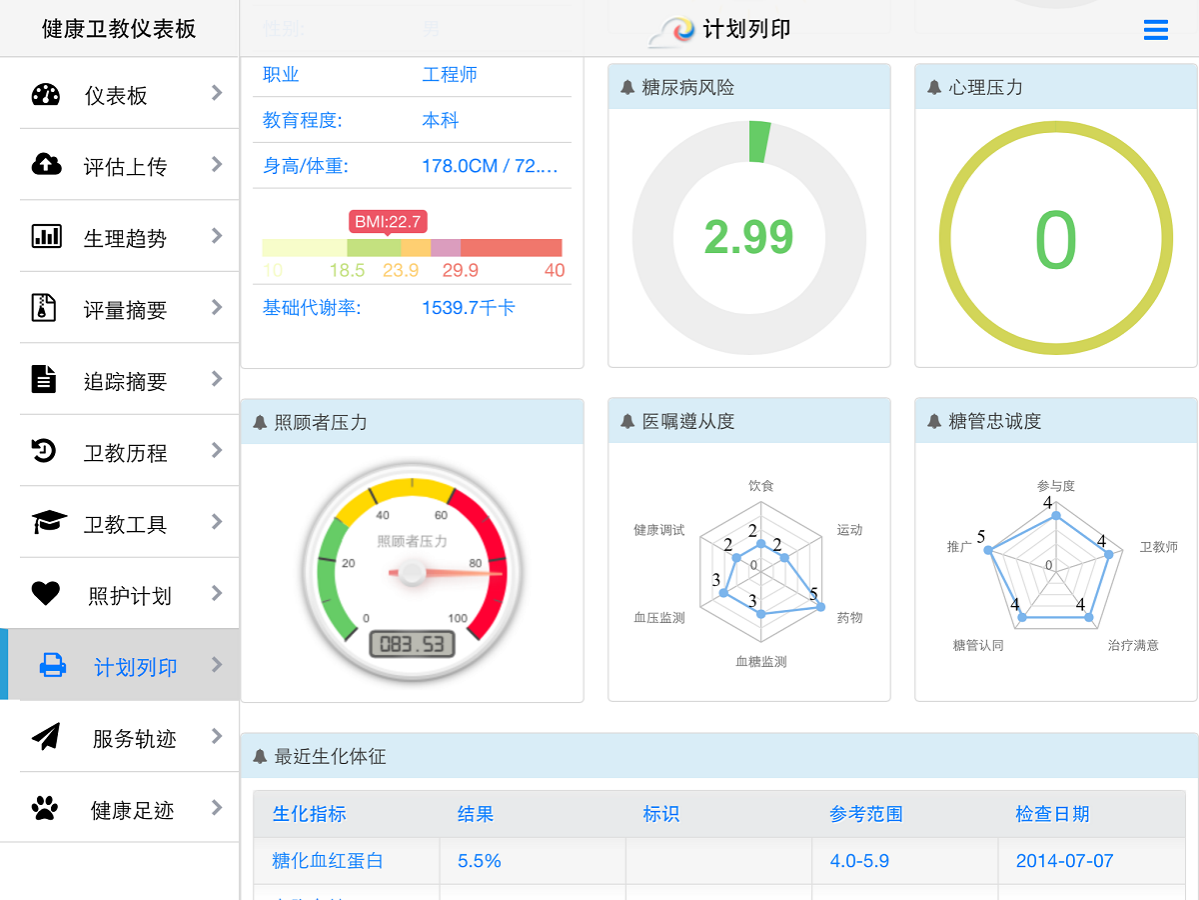
Interactive physician and patient engagement dashboard.

Patient clinical status, results from patient assessment, and readiness evaluation systems were presented on a physician dashboard. Patients interacted with the dashboard, and the system suggested an individualized education program based on evaluation results and patient lifestyle and preferences. Physicians and health educators used the comprehensive dashboard, wellness articles, and self-assessment tools to engage with patients. The dashboard tool illustrated the correlation between disease progression and patient’s self-efficacy (see Figure 8).

### Statistical Methods

The patient intervention activity and integrated data were recorded in the physician dashboard system. Data were analyzed using IBM SPSS Statistics version 19.0. A paired *t* test was used for pre- and postintervention assessment analysis, and the *t* test and chi-squared test were used for group comparisons.

## Results

In total, 121 patients (n=121) were enrolled in the study. The case group included 62 participants (case group, n=62; control group, n=59), and these patients completed 27,479 intervention interactions by using the IPMF. Patients lost to follow-up were eliminated; 118 patients completed postintervention assessment (case group, n=60; control group, n=58). A total of 97 patients completed both the preassessment and postassessment (case group, n=49, retention rate=82%; control group, n=48, retention rate=81%).

Although for the duration of follow-up, patients were free to choose categories of DM self-management according to their interests, there were no change requests during the patient engagement. The results of the case group were better than those of the control group, especially those for knowledge score, weight, and HbA_1c_ (*P*=.05; *P*=.03; *P*=.003). Participants at a high risk indicated high motivation to change and to achieve high scores in the self-care knowledge assessment (n=49, 95% CI −0.26% to −0.24%, *P*=.052; Table 1).

**Figure 9:**
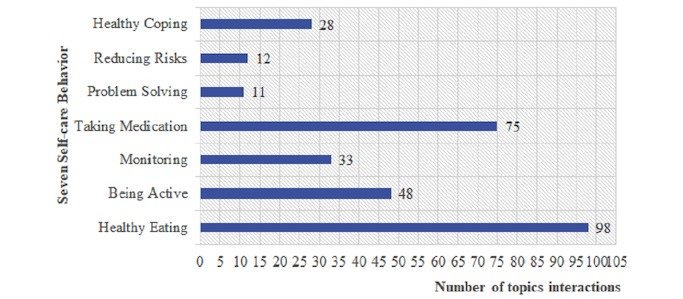
The participant engagement frequency by Diabetes self-management of 7 categories. Interactive response (n=305).

Of the 305 of interventions, healthy eating was selected 98 times (see Figure 9) and thus was the most-selected category (32%, 98/305), with the second most selected category being taking medication (25%, 75/305).

The demographic characteristics of case group patients who finished the behavioral education course and completed postintervention assessments (n=28) in high score and improved group are listed in Table 2. The average age was 63.71 years, and the age range was 37 to 88 years.

Among the participants, 75% reported a high school education level. Compliance frequency from pre- and postintervention-assessments were compared for analyzing the effects of the IPMF intervention (Table 3). The *P* values reflect differences between pre- and postintervention assessments, and results indicate that patients were willing to set a dietary goal for self-management (*P=*.04).

**Table 1:** Self-care knowledge assessment.

Self-knowledge and behavior change	Control group (n=48, 81%)			Case group (n=49, 82%)		
	Preassessment, mean (SD)	Postassessment, mean (SD)	*P* value between groups	Preassessment, mean (SD)	Postassessment, mean (SD)	*P* value between groups
Knowledge score	56.22 (10.97)	57.78 (10.68)	.33	60.16 (19.24)	65.08 (12.24)	.048
Weight (kg)	66.67 (17.28)	65.65 (16.98)	.07	67.86 (16.84)	57.5 (15.33)	.03
Body mass index (kg/m^2^)	25.29 (3.25)	24.94 (2.89)	.14	25.47 (3.31)	25.28 (2.93)	.23
Systolic blood pressure (mm Hg)	128.09 (17.36)	131.15 (18.24)	.57	130.24 (18.92)	131.37 (19.01)	.56
Diastolic bold pressure (mm Hg)	76.22 (12.11)	78.61 (12.98)	.52	74.99 (13.12)	75.58 (11.04)	.47
HbA_1c_^a^ (%)	8.95 (2.34)	7.82 (1.87)	.06	8.44 (2.28)	6.92 (1.27)	.03

^a^HbA_1c_: glycated hemoglobin.

**Table 2:** Demographic characteristics of participants (N=120).

Characteristic		n (%)
**Age (years)**		
	25≤years<40	1 (4)
	40≤years<50	3 (11)
	50≤years<60	6 (21)
	60≤years<70	9 (32)
	years≥70	9 (32)
**Gender**		
	Male	17 (61)
	Female	11 (39)
**Education**		
	No education	1 (4)
	Elementary school	6 (21)
	Junior high school	4 (14)
	High school	11 (39)
	College	5 (18)
	Graduate	1 (4)
**Occupation (n=120)**		
	Unemployed	8 (7)
	Public servant	5 (4)
	Office worker	21 (18)
	Businessman	17 (14)
	Retired	64 (53)
	Others	5 (4)

**Table 3:** Compliance comparison between pre- and postintervention assessments.

Compliance	Average (preassessment), mean (SD)	Average (postassessment), mean (SD)	*P* value
Dietary	3.5 (1.0)	3.8 (1.0)	.04
Exercise	3.0 (1.5)	3.3 (1.4)	.13
Medicine taking	4.6 (0.7)	4.4 (1.0)	.42
Blood glucose monitoring	3.2 (1.3)	3.4 (1.3)	.33
Blood pressure monitoring	3.2 (1.4)	3.2 (1.3)	.56
Health coping	3.8 (0.9)	4.1 (0.9)	.06

In addition to behavioral education compliance, the results of the Pearson chi-squared test indicate that patients’ average compliance frequencies increased after intervention (Table 4), with improvements in dietary compliance reaching statistical significance (*P=*.07). The study results indicate that other surveyed health behaviors, including smoking status and drinking status, did not affect dietary behavioral compliance.

**Figure 10:**
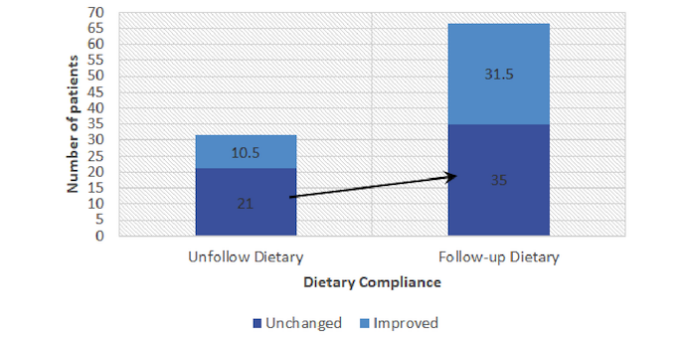
Compliance of improved patients among those using the intervention. Dietary response (n=98).

On the basis of the compliance changes, participants were then divided into an improved group (patients who exhibited improved compliance after the study) and an unchanged group (patients whose compliance was unchanged or decreased) for comparison. The improved group contained a higher number of patients who experienced diet-related intervention through the IPMF than the unchanged group (66.5% vs 31.5%; see Figure 10).

**Table 4:** Improved dietary compliance versus unchanged dietary compliance (n=16).

Behavioral education compliance (case group)		Dietary unchanged result, n (%)	Dietary improved result, n (%)	Pearson chi-squared *P* value
**Gender**				**.07**
	Male	12 (75)	5 (42)	—^a^
	Female	4 (25)	7 (58)	—
**Drinking status**				**.80**
	Nondrinker	7 (44)	4 (33)	—
	Occasional	3 (19)	2 (17)	—
	Daily drinker	6 (37)	6 (50)	—
**Mobile phone usage**				**.63**
	No	12 (75.0)	8 (67)	—
	Yes	4 (25.0)	4 (33)	—

^a^Not applicable.

**Figure 11:**
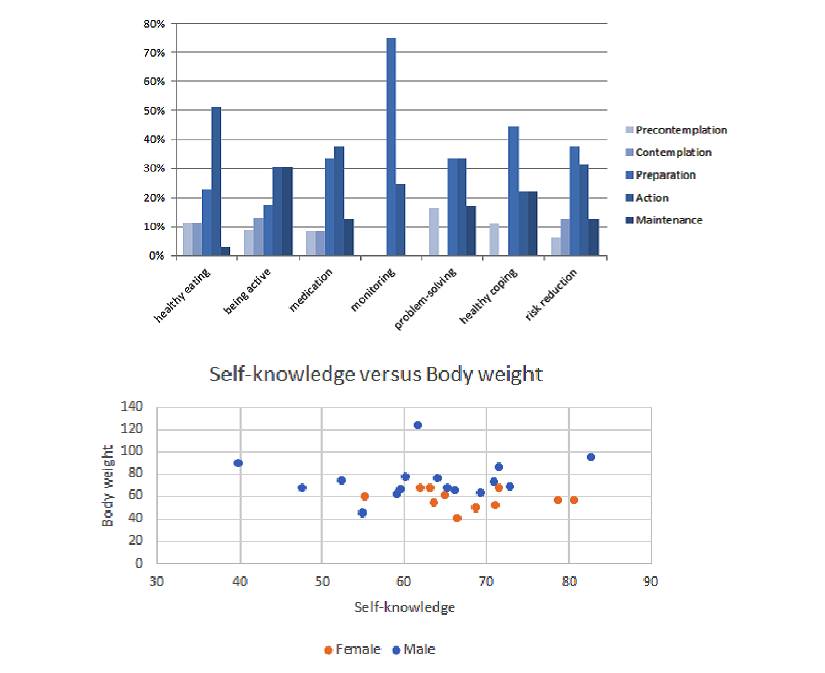
Intervention and self-behavior change (by transtheoretical model view).

According to behavioral change of TTM data on monitoring, patients could enter the preparation stage directly (precontemplation, contemplation, preparation, action, maintenance; 0%, 0%, 75%, 25%, and 0%, respectively) without needing blood glucose monitoring. For healthy eating, more time was required for the precontemplation, contemplation, and preparation stages, but the time required for the action stage was markedly higher (precontemplation, contemplation, preparation, action, maintenance; 11%, 11%, 23%, 52%, and 3%, respectively). In the overall behavior change period, male participants with higher body weights and body-mass indexes exhibited greater motivation to change. Compared with male participants, female participants exhibited higher scores in self-knowledge and self-care (see Figure 11).

## Discussion

### Principal Findings

This study indicates that overall patient compliance rate increased after the IPMF-based educational intervention [4,5,12,26]. Participants who completed postintervention assessments were older than 60 years. This finding demonstrates that age was not an impediment to mobile device usage.

The use of IPMF to improve patient compliance may help patient engagement and customer relationships and retain patients [12,13,33]. The personalized interactive education approach used in this study was associated with greater overall compliance with recommended health behaviors [1,19,27,34,35].

In addition, education level was not a barrier to using a mobile device (such as an iPad) for wellness education. Mobile phones are increasingly prevalent, but only a few participants owned a mobile phone. This low mobile phone ownership may have been correlated with average participant age [25].

Key aspects of self-efficacy for patients include the ability to use a mobile app to self-monitor progress and to be accountable for goals set [33]. In this study, the IPMF system may be particularly helpful for T2D patients with hypertension or who are overweight.

From another perspective, older people may encounter problems in engaging with interventions to reduce the risk of type 2 diabetes; the strong representation of this group in this study sample may be helpful for addressing relevant concerns [3]. This study indicated the participants were willing to join and complete the study regardless of whether they owned mobile phone, indicating that older patients can benefit from IoT resources [30]. This finding indicates that the intervention stimulated behavior changes in participants with greater interest in self-management.

### Strengths and Limitations

A strength of this study was the inclusion of individual personalized goals and an execution plan; this study was not treated as a research study. All patient feedback data came from the real world as opposed to being generated from a research setting.

A limitation inherent in mobile app interventions is that patient responses may differ from those of normal behavior. This analysis was limited after follow-up, and the findings may not be applicable to patients with type 2 diabetes with low motivation to use a mobile app for self-management.

### Implications

The results of this study indicated that persistent personal health management is correlated with positive feedback from physiological data [18,19]. In recent years, considerations of ethnic differences have served as the key element for providers designing treatment plans [7,16]. For example, investigating Chinese people’s lifestyles—including elements such as diet, exercise, and stress-coping strategies—requires a wide variety of data obtained from user feedback [9,39].

Developers must address and acknowledge barriers to the use of mobile app interventions, such as low computer literacy and low health maintenance motivation [5,21,33].

### Conclusions and Recommendation

This study found that (1) self-efficacy influences the factors of the IPMF intervention method and behavioral change model, and (2) a mobile app can be used to improve personalized care plans.

Despite the potential benefits of using a mobile app to support self-management of chronic diseases, we found that individual preferences can have a marked impact on personalized care plans and goal setting. As the mobile app for self-management in this study was developed and implemented rapidly, further work is necessary to produce a specific service that can enrich the quality of patient-centered care.

On the basis of this study, future related studies should consider the effects and continuity of behavioral changes resulting from interventions for enhancing self-efficacy. This research and the mobile interactive system will be revised and redeployed among an ethnic Asian population (eg, patients living in the United States with high motivation to use mobile apps to manage their personal health), and further evidence will be gathered to validate the effects of its use in health care self-management.

## Abbreviations

AADE7: American Association of Diabetes Educators 7 Self-Care Behaviors
DM: diabetes mellitus
HbA_1c_: glycated hemoglobin
IoT: Internet of Things
IPMF: interactive personalized management framework
T2D: type 2 DM
TTM: transtheoretical model
